# Antioxidant effect of spirulina in chronic periodontitis

**DOI:** 10.1097/MD.0000000000031521

**Published:** 2022-12-16

**Authors:** Vinay Rama Krishna Kaipa, Shaik Mohammed Asif, Khalil Ibrahim Assiri, Shahabe Abullais Saquib, Saeed Abdullah Arem, Suma Sree, Syed Mohammed Yassin, Mohammed Ibrahim, Mansoor Shariff, Shaik Mohammed Shamsudeen, Sultan Mohammed Kaleem, Abdul Ahad Ghaffar Khan

**Affiliations:** a Department of Periodontics, Mamata Dental College, Khammam, Telangana, India; b Department of Diagnostic Science and Oral Biology, College of Dentistry, King Khalid University, Abha, Kingdom of Saudi Arabia; c Department of Periodontics and Community Dental Sciences, College of Dentistry, King Khalid University, Abha, Saudi Arabia; d Department of Orthodontics, SVS Institute of Dental Sciences, Mahabubnagar, Telangana, India; e Department of Pediatric Dentistry and Orthodontic Sciences, College of Dentistry, King Khalid University, Abha, Saudi Arabia; f Department of Maxillofacial Surgery, College of Dentistry, King Khalid University, Abha, Kingdom of Saudi Arabia; g Department of Prosthetic Dentistry, College of Dentistry, King Khalid University, Abha, Kingdom of Saudi Arabia.

**Keywords:** chronic periodontitis, malondialdehyde, reactive oxygen species, spirulina

## Abstract

Production of reactive oxygen species (ROS) by polymorpho nuclear neutrophils occurs in chronic periodontitis. These ROS are mainly involved in bacterial destruction. However, extracellular release of same results in destruction of surrounding tissue. Spirulina, a potent antioxidant when administered sub gingivally in pockets can offer improved results in chronic periodontitis patients. This study was conducted to evaluate the effect of Spirulina when delivered sub gingivally in patients with chronic periodontitis.

**Methods:**

Sixty chronic periodontitis patients were divided into 2 groups. Group I (n = 30) were treated with Scaling and Root planning (SRP) followed by placement of placebo (SRP-P) and in Group II (n = 30) Spirulina microspheres were placed sub gingivally (SRP-S) following SRP. Clinical parameters like bleeding on probing (BOP), clinical attachment level (CAL), gingival index (GI) and probing pocket depth (PPD) were evaluated. Levels of salivary and serum Malondialdehyde (MDA) were estimated using ultra violet spectrophotometer.

**Results:**

At baseline, in both groups there was no statistically significant difference in clinical and biochemical parameters. Intra group comparisons of parameters from baseline to 90 days were statistically significant in both groups. After 90 days difference in clinical parameters and salivary MDA levels were statistically significant in SRP-S compared to SRP-P group. Though serum MDA levels were reduced in both groups, they were not significant statistically.

**Conclusions:**

Our study concluded that, local drug delivery of Spirulina adjunctive to SRP has potent antioxidant effect in treatment of chronic periodontitis.

## 1. Introduction

Periodontitis is a chronic inflammatory disease of surrounding and supporting structures of teeth. Periodontitis ramifies due to the loss of an intricate balance between microbial virulence factors and commensurate host response.^[1]^ Both non-surgical and surgical treatment modalities are utilized for treating periodontal disease. Suppression of putative periodontal pathogens can be done by mechanical therapy which constitutes scaling and root planning (SRP) and ultrasonic debridement.^[2]^ However, in case of deep or tortuous pockets, mechanical therapy alone might not eliminate anaerobic infection.^[3]^ Hence, to overcome this, administration of anti-microbials systemically and locally was instituted as an adjunct for SRP.^[4]^ Systemic antimicrobial therapy entails unwanted effects such as drug toxicity, development of resistant strains and drug interaction.^[5]^ Therefore, to overcome these flaws of systemic antibiotic therapy concept of local delivery of antimicrobial agents into periodontal pocket was developed. Local delivery of antimicrobials has inherent advantages like, drug being delivered to the base of the pocket at microbiologically efficacious concentration and sustaining concentration of the drug in pocket for a sufficient amount of time.^[6]^ Various local drug delivery systems for treating periodontitis are available of which microspheres are a variant. These microspheres are controlled drug release systems in form of drug-containing micro particles or microspheres, ranging from 10 to 500 microns in size, which are suspended in a carrier medium, and are capable of maintaining an effective drug concentration in periodontal pocket for a considerable period of time. Biodegradable as well as non-biodegradable materials were investigated for the microsphere preparation.^[7]^ Bio-degradable polylactide (PLA) or polylactide–glycolide (PLGA) has applications in protein/peptide drug delivery. Encapsulating these drugs in microspheres, helps maintain a higher constant value for prolonged time.^[8,9]^ Spirulina (Spirulina platenesis) is a photosynthetic cyanobacterium that is endowed with biological activity. It consists of many essential and non-essential macro and micronutrients that make it an ideal nutritional and dietary supplement.^[10]^ Chemical compounds like phenolic compounds, tocopherols, beta carotenes and phyocyanins show antioxidant properties. Phycocyanin a major constituent of Spirulina exists as a complex mixture of trimer and hexamer. It also possesses antioxidant property which explains its strong anti-inflammatory effect.^[11,12]^ In recent years, there is growing evidence regarding effect of oxidative stress (OS) on periodontal tissues by free radicals. OS develops due to disturbance of pro-oxidant-antioxidant balance in favor of the former. In oxygen-dependent pathway of host defences against bacteria, there is generation of reactive oxygen species (ROS) by polymorpho nuclear neutrophils which are basically engaged in destruction of bacteria. However, extracellular release of same results in surrounding tissue destruction.^[13]^ ROS has a very short life and is very difficult to detect their presence. Therefore, the final product of lipid peroxidation (LPO), that is, malondialdehyde (MDA), formed during oxidative degeneration, can be used to measure OS.^[14]^ MDA is as major biomarker that is present in saliva and serum which can be used to assess total antioxidant capacity (TAOC) of Spirulina.^[15]^ Therefore, this present study was conducted as a single-center, case control trial to evaluate antioxidant effect of Spirulina delivered sub gingivally in patients with chronic periodontitis. In addition, this study also aimed to assess effects of sub gingivally delivered Spirulina on various clinical parameters.

## 2. Methods

### 2.1. Study participants

This was a simple parallel randomized study conducted among 60 chronic generalized periodontitis patients between 35 and 65 years of age. The study followed the guidelines of the Declaration of Helsinki, study protocol was approved by the Human Ethics Committee (MDC16/05/20,257) and required permission was obtained. All methods were performed in accordance with the relevant guidelines and regulations given by institutional review board. An informed consent verbally and a written declaration were obtained from participants after discussing in detail about the purpose of the study.

### 2.2. Inclusion criteria

Subjects fulfilling following criteria were included in the study.

1. Chronic periodontitis patients with good systemic health.
2. Probing Pocket Depths (PPD) ≥ 4 mm and clinical attachment level (CAL) ≥ 4 mm.

### 2.3. Exclusion criteria

Subjects with 1 or more of the following conditions were excluded from study.

1. Aggressive periodontitis cases.
2. Pregnant females.
3. Smokers.
4. Patients who underwent periodontal therapy or any medication that would have influence on the periodontal tissues in preceding 6 months (cyclosporin, Aspirin).
5. Patients with any systemic condition or under any drug usage that would affect the salivary flow rate (oxybutynin, clozapine).

### 2.4. Procedure

Chronic periodontitis patients were selected based on the criteria given by Page and Eke (2007). Subjects with ≥ 2 areas with interproximal Clinical Attachment Loss of ≥ 4 mm, not on same tooth or ≥ 2 sites with interproximal Probing Depth ≥ 4 mm, not on same tooth were diagnosed as cases of chronic periodontitis.^[15]^ Randomly selected 60 subjects were divided into 2 groups of 30 each. only 1 site of each group was selected for the study (pre-molar or molar areas) with Clinical Attachment Loss of ≥ 4 mm and Probing Depth ≥ 4 mm. Group 1:30 patients with chronic periodontitis in whom after completing SRP a placebo was placed sub gingivally (SRP-P). Group 2:30 patients with chronic periodontitis in whom after completing SRP, local delivery of Spirulina was done sub gingivally (SRP-S).

Various clinical and biochemical parameters were assessed before and after the treatment.

Following clinical parameters were evaluated-

1. Gingival Index (GI) by Loe H and Silness P (1963).
2. Bleeding on Probing (BOP) using Sulcus Bleeding Index by Muhlemann HR and Son S (1971).
3. PPD evaluated using a (University of North Carolina) UNC-15 probe.
4. CAL evaluated using a UNC-15 probe.

Baseline (before SRP) and 3 months recording of clinical parameters were done. Single operator (operator A) treated both groups, and recording of all pre- and post-treatment clinical parameters were done by another examiner (operator B) who was blinded. Measurement of PPD and CAL were standardized by using a customized acrylic stent.^[16]^ and a UNC no. 15 color-coded probe. CAL were measured by calculating distance from apical extent of stent to the base of pocket and then subtracting distance from cemento-enamel junction to stent. Aqueous Spirulina solution was prepared from Spirulina powder (Spirulina^®^ Cellusyn Labs) and was dispersed in lipophilic organic continuous phase. A homogenous polymer solution was made by dissolving sodium alginate in purified water. Drug was then added and stirred thoroughly to form a thick dispersion which was later added drop wise through a syringe into solution of calcium chloride. Droplets were retained for fifteen minutes for curing reaction resulting in microsphere production. Microsphere were collected by decantation and obtained product was washed and dried for 24 hours at 45°C. Each microsphere contained a dosage of 2 mg of Spirulina, was injected in to pocket by using a syringe with blunt cannula. patients were instructed to refrain from chewing hard or sticky foods, brushing near the treated areas, or using any interdental aids for 1 week. Adverse effects were noted at recall visits. and any existent supra–gingival deposits were removed. Similarly, microspheres of placebo were prepared by using sodium alginate. MDA levels in saliva and serum were evaluated using colorimetric method. Salivary samples were collected according to the guideline of Navazesh et al^[17]^ Participants were asked to rinse their mouth with water 10 minutes before collection of saliva. Unstimulated saliva was collected by means of spitting method. Subjects were asked to swallow saliva prior to start of collection. Saliva was then allowed to accumulate in floor of the mouth by orofacial movements without stimulation of salivary glands. Subjects were asked to spit accumulated saliva into container. Samples were cooled at a temperature of 4°C and frozen (−20°C to −70°C) to the earliest. 5 mL of blood was collected from a large peripheral vein of into purple top Ethylene diamine tetra acetic acid tubes and centrifuged (2000 rpm) for 20 minutes. After centrifugation serum was pipetted into plastic containers with lid and stored at −20°C to −70°C until analyzed. MDA was resolved as thiobarbituric acid reactive substances (TBARS). Free MDA, as a measure of LPO, was spectrophotometrically estimated as TBARS after protein precipitation with Trichloroacetic acid. 500 µL of serum/saliva and 500 µL of saline were taken in test tube, and 1 mL of 24% trichloroacetic acid (TCA) was added. Centrifugation of mixture was done at 2000 rpm for twenty minutes. A milliliter supernatant was transferred into test tube to which 250 µL of thiobarbituric acid (TBA) reagent was added. Test tube was covered with a rubber cork and kept in water bath for 1 hour at 97°C followed by cooling under tap water. Contents were added to 500 µl of n-butanol and vortexed for 1 minute. Centrifugation of the mixture was done and superficial butanol layer was read at 532 nm under ultraviolet spectrophotometer. Values of unknown were derived from standard curve. Working calibrator was diluted according to yield standards with various concentrations for standard curve (Table 1).

**Table 1:** Procedure for standard preparation.

Additions	Blank	S1	S2	S3	S4	S5	S6
**Conc (µmol/L**)	-------	1.0	2.0	4.0	6.0	8.0	10.0
**Standard (µL**)	-------	100	200	400	600	800	1000
**Distilled water (µL**)	1000	900	800	600	400	200	------
**TBA (µL**)	250	250	250	250	250	250	250
**n-butanol**	500	500	500	500	500	500	500

µL = micro liter, µmol/L = micro molar per liter, Conc = concentration, S1 = standard one, S1 = standard one, S2 = standard two, S3 = standard three, S4 = standard four, S5 = standard five, S6 = standard six, TBA = ThioBarbituric acid.

### 2.5. Statistical analysis

Observed results were analyzed using Statistical Package for Social Science (SPSS 20) by IBM. A *P* value of < .05 was considered statistically significant. Inter-group comparisons were done using independent sample *t* test and intra-group comparisons were done using paired *t* test.

## 3. Results

Sixty patients with chronic periodontitis were randomly divided into 2 groups. Group I control group (SRP-P). Group II test group (SRP-S). All clinical parameters, that is, GI, bleeding index, PPD and CAL and biochemical parameters like MDA levels were evaluated from baseline to 3 months within the groups (intra group) (Tables 2 and 3). Inter group comparisons for clinical and biochemical parameters were made for both groups (Table 4). Mean value change in salivary MDA levels in SPR-P and SPR-S were 32.35 ± 15.38 and 45.71 ± 11.08 respectively, with a statistical significance and *P* value of .017 (Table 4). In relation to serum MDA levels mean values were 18.65 ± 27048 in SPR-P and 17.91 ± 20.22 in SPR-S which was statistically significant and with a *P* value of .004. Mean value in GI and BOP were 20.66 ± 29.90 and 23.91 ± 12.76 in SPR-P and 38.69 ± 14.62 and 50.21 ± 12.83 in SPR-S and both the indices had a statistical significance when SPR-P group was compared with SPR-S group with a similar *P* value of < .001 (Table 4). Mean value in PPD in SPR-P group was 6.66 ± 15.94 and 39.85 ± 9.01 in SPR-S group with *P* value < .001 which was statistically significant. Similarly, CAL had 7.46 ± 12.61 and 31.53 ± 8.95 in SPR-P and SPR-S groups respectively, with a statistically significant *P* value < .001 (Table 4).

**Table 2:** Comparison of mean values of various parameters in SRP-P group at baseline and 90 days.

SRP-P	Baseline		90 d		*P* value
	Mean	SD	Mean	SD	
**Salivary MDA**	0.34	0.05	0.23	0.05	.05*
**Serum MDA**	3.86	1.31	3.14	0.88	<.001**
**GI**	2.42	1.02	1.92	0.25	.017*
**BOP**	2.30	0.27	1.75	0.30	<.001**
**PPD (mm**)	3.90	0.86	3.64	0.70	.036*
**CAL (mm**)	4.15	0.99	3.84	0.73	.011*

BOP = bleeding on probing, CAL = clinical attachment loss, GI = gingival index, MDA = malondialdehyde, PPD = probing pocket depth, SD = standard deviation, SRP-P = scaling root planning with placebo.

*P* < .05 is statistically significant.

* significant.

** highly significant.

**Table 3:** Comparison of mean values of various parameters in SRP-S group at baseline and 90 days.

SRP-S	Baseline		90 days		*P* value
	Mean	SD	Mean	SD	
**Salivary MDA**	0.35	0.05	0.19	0.04	<.001*
**Serum MDA**	4.13	1.03	3.39	0.97	<.001*
**GI**	2.30	0.40	1.41	0.41	<.001*
**BOP**	2.29	0.34	1.14	0.33	<.001*
**PPD (mm**)	4.09	0.78	2.46	0.82	<.001*
**CAL (mm**)	4.44	0.93	3.04	0.99	<.001*

BOP = bleeding on probing, CAL = clinical attachment loss, GI = gingival index, MDA = malondialdehyde, PPD = probing pocket depth, SD = standard deviation, Sig = significant, SRP-S = scaling root planning with spirulina.

*P* < .05 is statistically significant.

* highly significant.

**Table 4:** Comparison of mean percentage changes in various parameters in SRP-P and SRP-S groups.

Percentage	Group				*P* value
	SRP-P		SRP-S		
	Mean	SD	Mean	SD	
Salivary MDA	32.35	15.38	45.71	11.08	.017*
Serum MDA	18.65	27.48	17.91	20.22	.004**
GI	20.66	29.90	38.69	14.62	<.001**
BOP	23.36	12.76	50.24	12.83	<.001**
PPD (mm)	6.66	15.94	39.85	9.01	<.001**
CAL (mm)	7.46	12.61	31.53	8.95	<.001**

BOP = bleeding on probing, CAL = clinical attachment loss, GI = gingival index, MDA = malondialdehyde, PPD = probing pocket depth, SD = standard deviation, SRP-P = scaling root planning with placebo, SRP-S = scaling root planning with spirulina.

*P* < .05 is statistically significant.

* significant.

** highly significant.

## 4. Discussion

**Figure 1.**
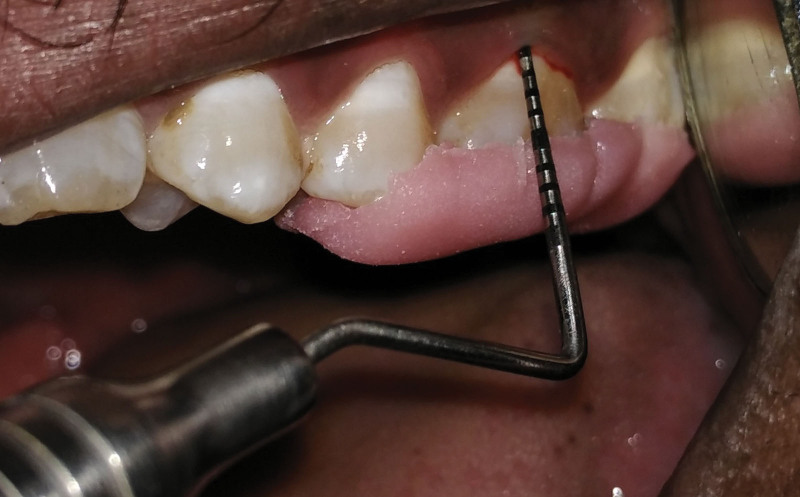
Probing pocket depth at baseline SRP-P group. SRP-P = scaling and root planning-placebo.

**Figure 2.**
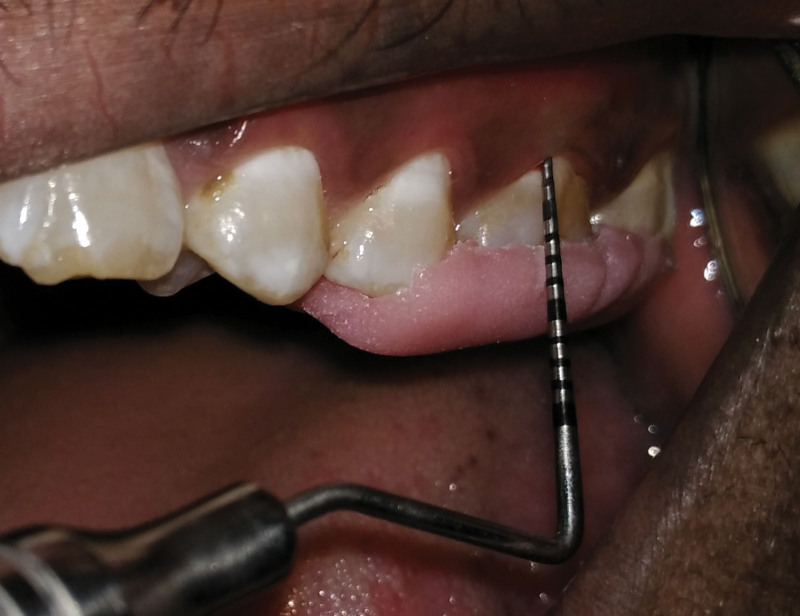
Probing pocket depth after 90 days SRP-P group. SRP-P = scaling and root planning-placebo.

**Figure 3.**
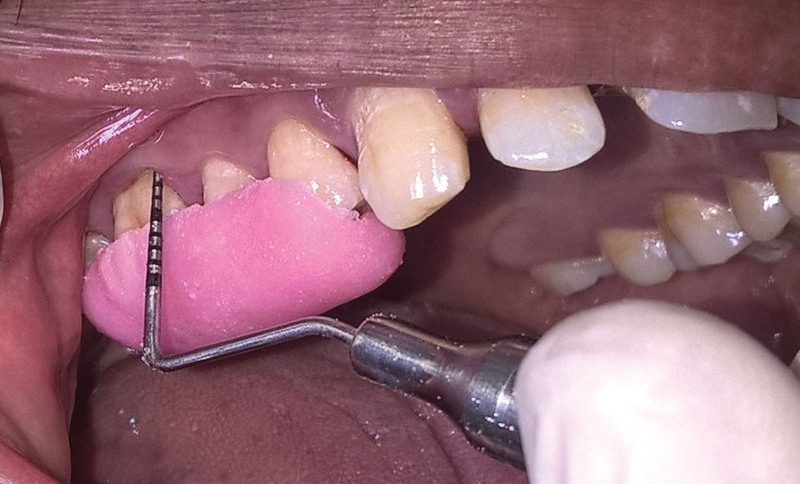
Probing pocket depth at baseline SRP-S group. SRP-S = scaling and root planning-spirulina.

**Figure 4.**
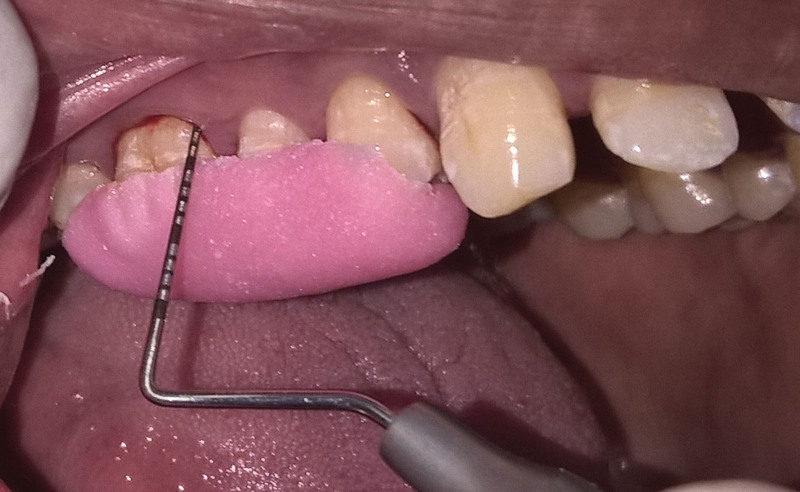
Probing pocket depth after 90 days SRP-S group. SRP-S = scaling and root planning-spirulina.

Periodontitis is characterized by inflammation, pocket formation, loss of fibrous attachment and destruction of alveolar bone. They are widely accepted as being caused by bacteria associated with dental plaque.^[1]^ Loe H and Bakdash stated that plaque removal remains primary and most widely accepted means of controlling supra gingival plaque and maintaining gingival health.^[2,18]^ SRP is considered as a gold standard in treatment of periodontal disease.^[19]^ It is not only during initial phase of treatment but also as a maintenance therapy to prevent recurrence.^[20]^ Antioxidant activity of Spirulina is due to presence of 2 phycobiliproteins that is phycocyanin and allophycocyanin. Its activity was found to be proportional to the concentration of phycobiliproteins which was mainly due to content of phycocyanin.^[21]^ Many toxicological studies have proven Spirulina’s safety. In a study by Naif Abdullah, Spirulina was evaluated for concentration of 6 typical heavy metals/minerals (Nickel, Zinc, Mercury, Platinum, Magnesium, and Manganese). It was concluded that concentration of inorganic elements was not found to exceed the regulatory levels, and Spirulina was considered as safe food.^[22]^ It is also considered as safe by US Food and Drug Administration under generally recognized as safe category.^[23–25]^ Mahendra J et al in 2013 assessed clinical efficacy of Spirulina gel adjunctive to SRP. Their study concluded that Spirulina has potent anti-inflammatory effect due to its strong anti-oxidant activity and less toxicity.^[12]^ MDA levels can be monitored by LPO which is a result of OS.^[26–28]^ LPO is an inflammatory process that starts via the oxidation of polyunsaturated fatty acids and is maintained through chain reactions, resulting in the synthesis of MDA, and is commonly used as an index of peroxidation.^[29]^ Present study was conducted to evaluate antioxidant effect of sub gingivally delivered Spirulina in patients with chronic generalized periodontitis. Spirulina was locally delivered as microspheres and its antioxidant effect were measured by using clinical and biochemical parameters. Carton et al^[30]^ Kaldahl et al^[31]^ Dahle’n et al^[32]^ recommended as follow up period of 90 days. It is because, maximum reduction in probing depth and clinical attachment gain occurs within 1 to 3 months of SRP. Albeit recuperation of periodontium occurs over 9 to 12 months. Therefore, assessment of treatment response should be performed not less than 4 weeks of therapy. In both SRP-S and SRP-P groups there was a significant improvement in GI, BOP, PPD and CAL levels from baseline to 90 days. In SRP-P group the mean reduction in the GI from baseline to 90 days was statistically significant (*P* = .017). BOP values also reduced significantly (*P* < .001) from baseline to 90 days. Changes in mean reduction of PPD and gain in CAL from baseline to 90 days was statistically significant (Table 2) (Figs. 1 and 2). Our results are in accordance with study conducted by Haffajee et al^[33]^ MarijaIvie-Kardum et al^[20]^ A statistically significant reduction was noticed in all clinical parameters. In SRP-S group, there was a significant reduction in clinical parameters viz GI, BOP, PPD and CAL from baseline to 90 days (Figs. 3 and 4) (Table 3). Our results are in accordance with study conducted by Mahendra et al where there was a significant reduction in PPD and CAL 120 days after the placement of Spirulina gel sub gingivally.^[12]^ Spirulina inhibits nuclear transcription factor activation which is in turn activated by ROS, with concomitant induction and expression of various cytokines and enzymes which are involved in the initiation and progression of inflammatory diseases.^[34]^ Mean reduction in GI value was higher and statistically significant in SRP-S group when compared to SRP-P with a mean percentage change variation from 20.66 in placebo group to 38.69 in Spirulina group (*P* < .001) (Table 4). BOP mean values reduced significantly in SRP-S group compared to SRP-P with a mean percentage change of 23.36 in placebo and 50.24 in Spirulina group (*P* < .001) (Table 4). Results of present study are in concurrence with study conducted by Madhu Bhatia et al on 1% curcumin gel, a potent antioxidant as local delivery.^[35]^ Reduction in BOP, PPD and improvement in CAL were seen in group with SRP and curcumin compared with SRP alone group over a follow up 3 months and 6 months. In present study mean changes in PPD and CAL were statistically significant (*P* < .001) (Table 4) in SRP-S group when compared to SRP-P group. Miranda MS et al in a study suggested that methanolic extract of Spirulina has antioxidant compounds which prevent oxidation when absorbed.^[36]^ These changes were in accordance to study conducted by Mahendra J et al using Spirulina gel.^[12]^ There was a significant difference between mean salivary MDA (*P* = .05) and serum MDA (*P* < .001) levels from baseline to 90 days in the SRP-P group (Table 2). Our results were in accordance with study conducted by J Khalili et al in which salivary MDA levels of 104 individuals were compared with healthy controls.^[37]^ A significant increase in MDA levels were noticed in patients compared to control group. They concluded that, salivary MDA level detection provided additional advantages in elucidating periodontal disease pathogenesis. In a study conducted by D Wei et al on LPO levels, total oxidant status and superoxide dismutase in serum, saliva and gingival crevicular fluid (GCF) in chronic periodontitis patients before and after periodontal therapy showed no much significant change in serum and salivary MDA levels.^[38]^ However, their results are contradictory to results obtained in present study. Fatemeh Ahmadi-Motamayel et al estimated salivary and serum MDA, and OS levels in chronic periodontitis patients.^[39]^ They concluded that, salivary MDA and serum MDA levels were elevated in chronic periodontitis patients. Results obtained in their study are in accordance with our study. In SRP-S group, there was a significant (*P* < .001) reduction in salivary and serum MDA levels from baseline to 90 days (Table 3). Results of our study are in accordance with study conducted by Miranda et al where, Spirulina showed a significant antioxidant effect in both in vitro and in vivo conditions.^[36]^ Spirulina extract dramatically inhibits production of TBARS that is MDA to 95%, indicating potent antioxidant activity of Spirulina. However, there are no studies available in literature to compare efficacy of sub gingivally delivered Spirulina on serum MDA levels. Studies relating to antioxidant effect of Spirulina when administered orally are available, which would not be apt to compare with our study, where Spirulina is delivered sub gingivally. Wan-Loy et al assessed antioxidant effect of aqueous extract obtained from Spirulina and its protective effect on free radical induced cell death. Assessment was done using chemical methods and cell-based assays. Their study concluded that, Spirulina extract has a protective effect against free radical induced apoptosis.^[40]^ Mean reduction in salivary MDA levels in SRP-S group were higher and statistically significantly when compared to MDA levels in the SRP-P group. Mean percentage changes in salivary MDA levels in SRP-P and SRP-S group were 32.35 and 45.71 respectively and were statistically significant (*P* = .017). The mean percentage changes in serum MDA levels in SRP-S and SRP-S groups were 18.65 and 17.91 respectively, which are significant statistically (*P* = .004) (Table 4). To our knowledge, there have been no studies reported to know effect of sub gingivally delivered Spirulina microspheres on salivary and serum MDA levels. Hence, a direct comparison with other studies to evaluate antioxidant effect on biochemical parameters was not possible. Medline search reveals no comparative study relating to results of our present study. Exclusive findings of this study are directly related to the antioxidant effect of sub-gingivally delivered Spirulina in chronic periodontitis patients. Therefore, further studies with larger sample size, longer reevaluation periods and increased follow-up are required for better evaluation. Within its limits, this study supports the role of Spirulina as a potent antioxidant in chronic periodontitis treatment when delivered sub gingivally. Further studies are required to substantiate this role of Spirulina.

Present study evaluated antioxidant effect of sub-gingivally delivered Spirulina in chronic periodontitis patients. Amount of reduction was significantly higher when Spirulina was used as local drug delivery. Clinical parameters and salivary MDA were reduced significantly in both groups, indicating that salivary antioxidant levels rather than serum antioxidant levels exhibit a correlation with the clinical measurements. Thus, from this study it can be concluded that, local drug delivery of Spirulina adjunctive to SRP has a potent antioxidant effect in treatment of chronic periodontitis. However, long term studies are required to establish Spirulina as a reliable drug for local delivery in the treatment of chronic periodontitis.

## Author contributions

**Conceptualization:** Shaik Mohammed Asif, Vinay Rama Krishna Kaipa.

**Data curation:** Suma Sree, Khaleel Ibrahim Assiri.

**Formal analysis:** Shahabe Abullais Saquib.

**Investigation:** Syed Mohammed Yassin, Saeed Abdullah Arem.

**Methodology:** Syed Mohammed Yassin, Saeed Abdullah Arem.

**Project administration:** Vinay Rama Krishna Kaipa.

**Resources:** Mansoor Shariff, Mohammed Ibrahim.

**Software:** Shaik Mohammed Asif, Shaik Mohammed Shamsuddin.

**Supervision:** Vinay Rama Krishna Kaipa, Mohammed Ibrahim.

**Validation:** Suma Sree, Khaleel Ibrahim Assiri, Sultan Mohammed Kaleem, Abdul Ahad Ghaffar Khan.

**Visualization:** Mansoor Shariff, Sultan Mohammed Kaleem, Abdul Ahad Ghaffar Khan.

**Writing – original draft:** Shaik Mohammed Asif, Vinay Rama Krishna Kaipa.

**Writing – review & editing:** Mohammed Ibrahim, Mansoor Shariff.
